# A case of multiple abscesses caused by *Mycobacterium mageritense* after salvage esophagectomy for esophageal cancer

**DOI:** 10.1093/jscr/rjaf111

**Published:** 2025-12-18

**Authors:** Kohei Takura, Koshiro Ishiyama, Shota Igaue, Ryoko Nozaki, Daichi Utsunomiya, Yuto Kubo, Kentaro Kubo, Daisuke Kurita, Junya Oguma, Mika Shiotsuka, Osamu Kobayashi, Hiroyuki Daiko

**Affiliations:** Department of Esophageal Surgery, National Cancer Center Hospital, 5-1-1 Tsukiji, Chuo-ku, Tokyo 104-0045, Japan; Department of Esophageal Surgery, National Cancer Center Hospital, 5-1-1 Tsukiji, Chuo-ku, Tokyo 104-0045, Japan; Department of Esophageal Surgery, National Cancer Center Hospital, 5-1-1 Tsukiji, Chuo-ku, Tokyo 104-0045, Japan; Department of Esophageal Surgery, National Cancer Center Hospital, 5-1-1 Tsukiji, Chuo-ku, Tokyo 104-0045, Japan; Department of Esophageal Surgery, National Cancer Center Hospital, 5-1-1 Tsukiji, Chuo-ku, Tokyo 104-0045, Japan; Department of Esophageal Surgery, National Cancer Center Hospital East, Chiba, Japan; Department of Esophageal Surgery, National Cancer Center Hospital, 5-1-1 Tsukiji, Chuo-ku, Tokyo 104-0045, Japan; Department of Esophageal Surgery, National Cancer Center Hospital, 5-1-1 Tsukiji, Chuo-ku, Tokyo 104-0045, Japan; Department of Esophageal Surgery, National Cancer Center Hospital, 5-1-1 Tsukiji, Chuo-ku, Tokyo 104-0045, Japan; Office of Infection Control and Prevention, National Cancer Center Hospital, Tokyo, Japan; Office of Infection Control and Prevention, National Cancer Center Hospital, Tokyo, Japan; Department of Esophageal Surgery, National Cancer Center Hospital, 5-1-1 Tsukiji, Chuo-ku, Tokyo 104-0045, Japan

**Keywords:** *Mycobacterium mageritense*, tuberculous mycobacterial infection, esophagectomy, esophageal cancer, salvage surgery

## Abstract

A 54-year-old Japanese male, previously diagnosed with esophageal cancer (cT1N1M0, cStage I), underwent robot-assisted thoracoscopic subtotal salvage esophagectomy to achieve non-CR after chemoradiation. On the 65th postoperative day, the patient experienced persistent fever and elevated C-reactive protein levels with an unclear cause. A computed tomography scan revealed multiple abscesses throughout the body, including in the dorsal trachea. Drainage treatment was performed, and *Mycobacterium mageritense* was identified in mycobacterial cultures. The patient responded well to treatment with amikacin, imipenem/cilastatin, linezolid, and levofloxacin. Postoperative infections following esophagectomy are often caused by oral bacteria, such as Streptococcus species. This report highlights a rare case of a post-salvage esophagectomy patient who developed multiple abscesses due to *M. mageritense*. In patients with unexplained postoperative symptoms, consideration of nontuberculous mycobacterial infections may be important.

## Introduction

Esophagectomy is a highly invasive surgical procedure that can result in severe complications, potentially leading to fatal outcomes. Vigilance is crucial for monitoring complications such as anastomotic leak, pneumonia, and recurrent laryngeal nerve palsy [1]. These complications are often suspected when postoperative fever occurs, prompting bacterial and fungal cultures and treatment with broad-spectrum antibiotics. Although uncommon, nontuberculous mycobacterial infections may arise post-surgery. *Mycobacterium mageritense*, identified as a new nontuberculous mycobacterial species in 1997, exhibits rapid growth characteristics [2]. Although *M. mageritense* has been observed in various clinical settings, its link to surgical procedures is exceedingly rare. Herein, we describe a patient presenting with unexplained fever and inflammation after the 65th day of salvage esophagectomy who was diagnosed with multiple abscesses caused by *M. mageritense*, a nontuberculous mycobacterial infection.

## Case report

**Figure 1:**
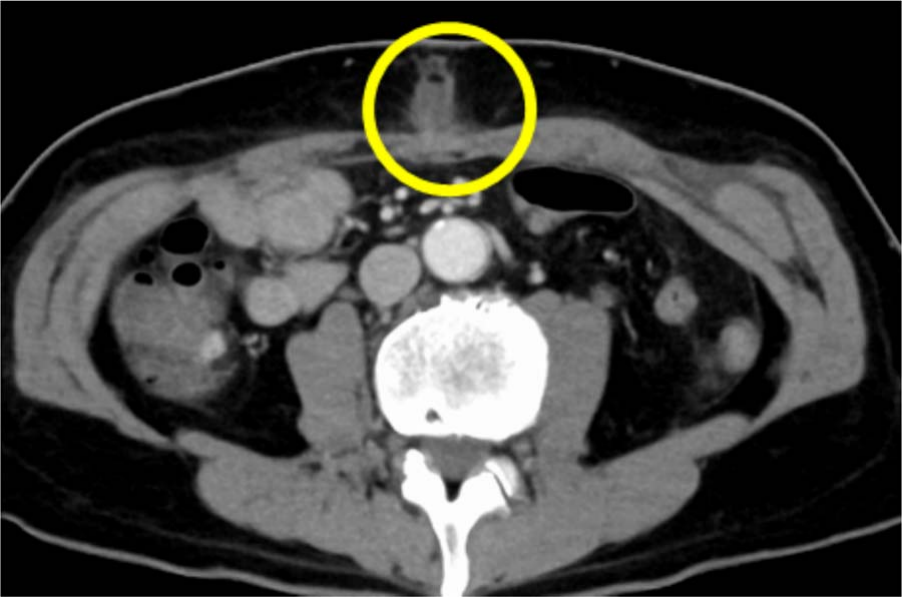
CT findings suggestive of an SSI. The circles indicate abscesses.

A 54-year-old Japanese male patient, previously healthy, was diagnosed with esophageal cancer (cT1N1M0, cStage I). He underwent radical chemoradiotherapy (FP-RT, 50.4 Gy/28Fr); however, the primary lesion persisted. Subsequently, he underwent a robot-assisted thoracoscopic subtotal salvage esophagectomy lasting for 9 h and 24 min, with a blood loss of 130 mL. Postoperative pathological examination revealed ypT2N3M0, ypStage IV A. The patient developed suspected pneumonia on postoperative day one and was treated with antibiotics. Despite a change in antibiotics, his fever persisted, prompting further investigation and treatment adjustments. Although there were no overt signs of cough or pneumonia, covert aspiration pneumonia was considered, and treatment with broad-spectrum antibiotics was initiated on postoperative day 14. Tests for various collagen disease markers and cytomegalovirus antigens revealed negative results, ruling out infectious endocarditis. Despite the negative cultures, the patient’s fever persisted without improvement in C-reactive protein levels. Regular blood cultures were consistently negative. Sputum cultures were also performed, revealing only common oral flora. Consequently, treatment with broad-spectrum antibiotics was continued. On postoperative day 24, the patient developed surgical site infection (SSI) at the midline incision (Fig. 1). Wound debridement was performed, resulting in a gradual decrease in fever frequency; the patient was discharged thereafter.

**Figure 2:**
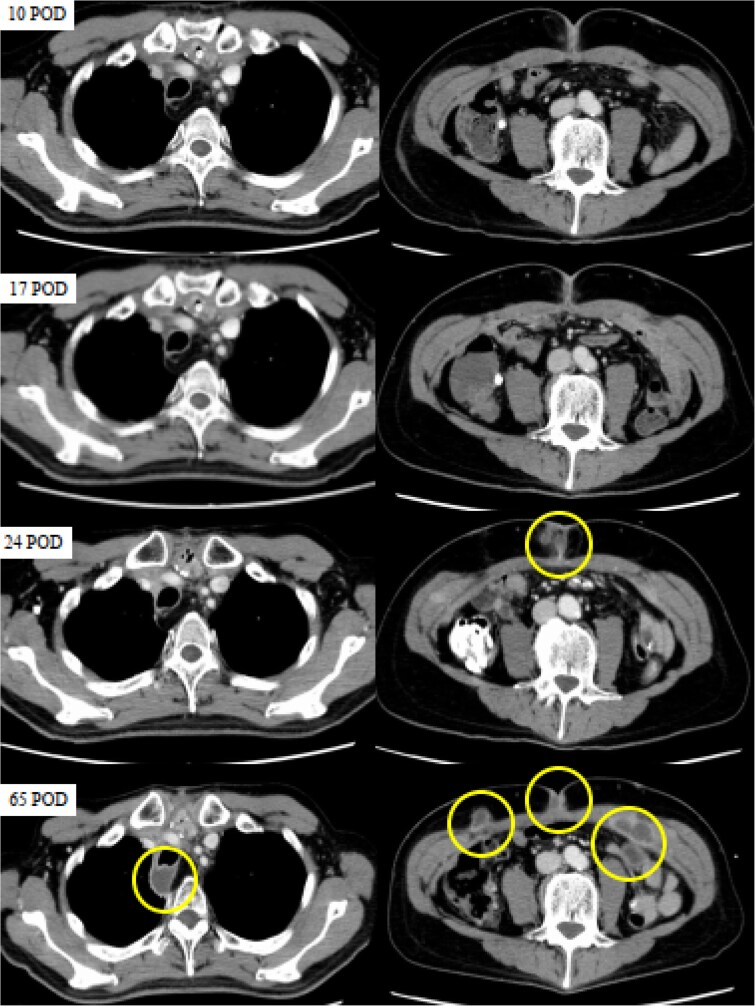
CT images obtained on postoperative days (POD) 10, 17, 24, and 65. The circles indicate abscesses. CT scans were performed on POD 10 and 17 to investigate the high levels of inflammation, but no abnormalities were detected. While no apparent abnormalities were observed on POD 10 and 17, SSI was evident on POD 24, and multiple abscesses had formed in the posterior trachea and abdominal wall by POD 65.

**Figure 3:**
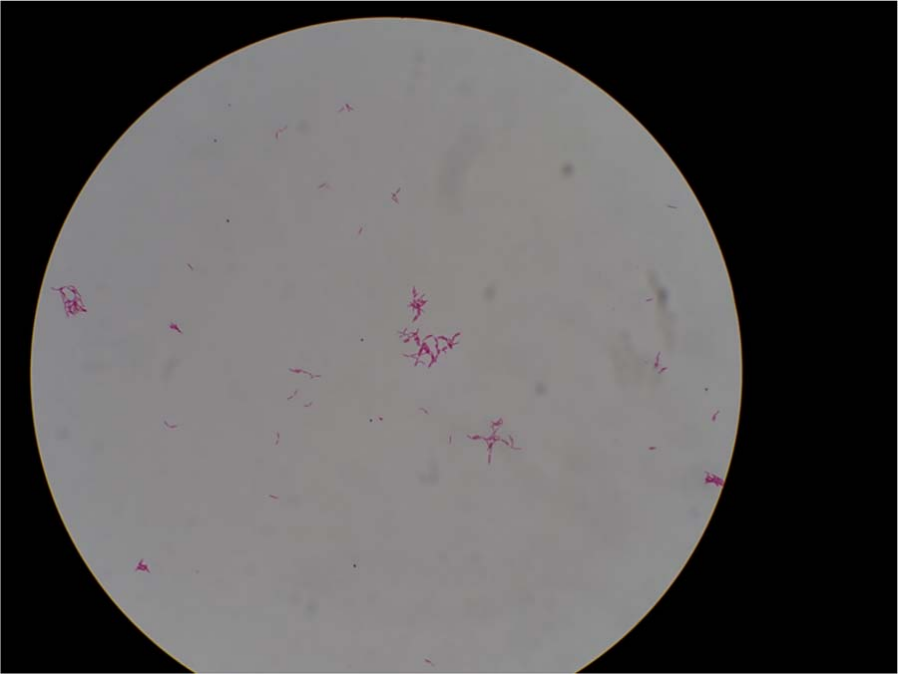
Acid-fast bacilli identified by Ziehl–Neelsen staining of liquid culture. Magnification, ×1000.

However, a computed tomography (CT) scan performed during an outpatient follow-up on postoperative day 65 revealed multiple abscesses, prompting the patient’s readmission for further management. The abscesses were drained percutaneously; additional cultures, including mycobacterial testing, were conducted to identify the cause. Although the general bacterial and fungal cultures were negative, the mycobacterial cultures were positive on postoperative day 80 (Fig. 2). The general bacterial and fungal cultures were negative; however, on postoperative day 80, the mycobacterial cultures were positive (Fig. 3). Following wound debridement, fever frequency gradually decreased; the patient’s condition improved steadily.

**Figure 4:**
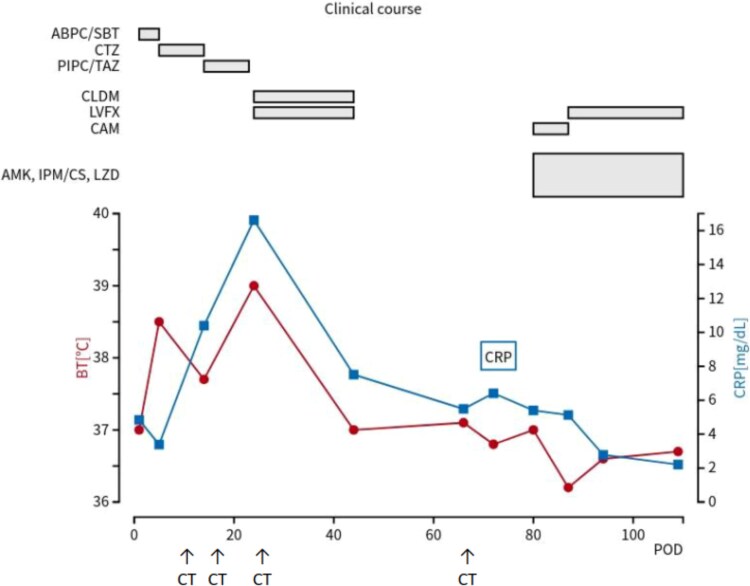
Clinical course of the patient. The red line represents body temperature, and the blue line indicates CRP levels. The upper section shows the timeline of antibiotics administered. CRP, C-reactive protein; POD, postoperative day; BT, body temperature; ABPC/SBT, ampicillin/sulbactam; CAZ, ceftazidime; PIPC/TAZ, piperacillin/tazobactam; LVFX, levofloxacin; CLDM, clindamycin; AMK, amikacin; IPM/CS, imipenem/cilastatin; LZD, linezolid; CAM, clarithromycin.

A nontuberculous mycobacterial infection was confirmed, and treatment was initiated with multiple antibiotics. The pathogen was identified as *M. mageritense*, leading to targeted therapy adjustments based on drug susceptibility. Based on drug susceptibility test results on postoperative day 87, amikacin and imipenem/cilastatin were discontinued (Table 1). Due to multiple postoperative complications, including pneumonia, SSI, and nontuberculous mycobacterial infection, the patient required prolonged hospitalization for effective treatment and management. He was ultimately discharged on postoperative day 112 after significant clinical improvement (Fig. 4).

**Table 1:** Minimum inhibitory concentrations (MICs) of various antibiotics tested against *Mycobacterium mageritense*

Antibiotics	MIC
Amikacin	32
Tobramycin	>16
Imipenem/Cilastatin	≤2
Faropenem	4
Levofloxacin	≤1
Moxifloxacin	≤0.25
Azithromycin	>64
Clarithromycin	>64
Sulfamethoxazole and Trimethoprim	76
Doxycycline	>16
Meropenem	4
Linezolid	2
Clofazimine	1
Sitafloxacin	≤0.25

MICs of different antibiotics. No resistance to levofloxacin and linezolid was observed.

## Discussion

In this case, we encountered an extremely rare infection of *M. mageritense* following a robot-assisted thoracoscopic subtotal salvage esophagectomy. *M. mageritense* is a nontuberculous mycobacterial species identified as a new species of rapidly growing mycobacteria (RGM) in 1997, distinct from the *Mycobacterium smegmatis* group [2]. RGM belong to Runyon’s Group IV and are present in environments such as soil and water, with the ability to cause infections in the skin and soft tissues [3, 4]. Furthermore, medical-related infections have been reported. Previously reported medical-related infections by *M. mageritense* include those associated with programmed cell death 1 (PD-1)-related immune-related adverse events (irAEs) [5], catheter-associated bloodstream infections [6], and infections in immunocompromised patients [7, 8]. However, the link between *M. mageritense* and surgery-related infections has not been widely recognized or documented [9–12].

In addition to our case, a few other cases of *M. mageritense* infections have been reported. For instance, one report described a case of SSI following orthopedic surgery, wherein *M. mageritense* was isolated from contaminated surgical instruments [13]. Another study reported a catheter-associated bloodstream infection in an immunocompromised patient undergoing chemotherapy [6]. Although these cases highlight the opportunistic nature of *M. mageritense*, reports of infections directly linked to esophageal surgery remain exceedingly rare.

In this case, the patient was a young male with esophageal cancer who had undergone chemoradiotherapy, which may have compromised his immune system. However, he had not been exposed to environmental factors such as soil or water, nor had he experienced any significant trauma before the surgery. Various factors may have contributed to the onset of *M. mageritense* infection. Despite being a minimally invasive robot-assisted procedure, salvage surgery for esophageal cancer involves a high level of invasiveness [14], which could potentially compromise the patient’s perioperative immune system. Additionally, there have been documented cases in the medical literature on RGM, including *M. mageritense*, contaminating surgical instruments [13]. Therefore, it is conceivable that the patient contracted *M. mageritense* from contaminated surgical instruments during or after the surgery. This initial infection likely led to an SSI; subsequently, multiple systemic abscesses due to bloodstream dissemination developed. Such rare cases highlight the pathogenic potential of *M. mageritense* in surgical settings, underscoring the critical importance for infection prevention measures.

In our case, the diagnosis of *M. mageritense* infection was confirmed 80 days after surgery. The significant delay in diagnosis resulted from the initial focus on excluding more common infections and collagen diseases, inadvertently overlooking mycobacterial infection as a potential differential diagnosis. This highlights the importance for considering RGM infections in cases of persistent postoperative fever and elevated inflammatory markers. Prompt initiation of mycobacterial cultures, alongside general bacterial and fungal cultures, could facilitate timely diagnosis and improve patient outcomes. This emphasizes the significance of considering mycobacterial infection in cases of persistent, unexplained fever, and elevated inflammatory markers post-surgery, highlighting the need for promptly conducting mycobacterial cultures.

In cases involving persistent unexplained fever and high inflammation levels following highly invasive surgeries, such as salvage esophagectomy, clinicians should consider performing mycobacterial cultures early in the diagnostic process. This should be done alongside routine bacterial cultures, fungal cultures, and tests for connective tissue diseases. Additionally, strict sterilization protocols for surgical instruments and heightened awareness of potential RGM contamination in the surgical environment may help reduce the risk of such infections.
